# The Roles and Challenges of Advanced Therapies in the Management of Refractory Immune Thrombocytopenia: A Case Report and Review of the Literature

**DOI:** 10.7759/cureus.36146

**Published:** 2023-03-14

**Authors:** Beatrice E Torere, Henry O Aiwuyo, Joseph Weigold, Gene Gerlach, Nosakhare Ilerhunmwuwa, Usman Khan, Tatiana Belousova

**Affiliations:** 1 Internal Medicine, North Mississippi Medical Center, Tupelo, USA; 2 Internal Medicine, Brookdale University Hospital Medical Center, Brooklyn, USA; 3 Hematology and Oncology, North Mississippi Medical Center, Tupelo, USA; 4 Pathology, North Mississippi Medical Center, Tupelo, USA

**Keywords:** splenomegaly, intraparenchymal hemorrhage, splenectomy, immune thrombocytopenia, refractory immune thrombocytopenia

## Abstract

The management of primary immune thrombocytopenia (ITP) is becoming a subject of interest as there appears to be treatment failure and resistance to modern conventional treatment, necessitating a more universal and goal-directed approach to management. Our patient is a 74-year-old male who was diagnosed with ITP six years ago and recently presented to the emergency department (ED) with complaints of melena stools and severe fatigue lasting for two days. Prior to the ED presentation, he had received multiple lines of treatment including splenectomy. On admission, the pathology after splenectomy showed a benign enlarged spleen with a focal area of intraparenchymal hemorrhage/rupture and changes compatible with ITP. He was managed with multiple platelet transfusions, IV methyl prednisone succinate, rituximab, and romiplostim. His platelet counts improved to 47,000, and he was discharged home on oral steroids with outpatient hematology follow-up. However, in a few weeks, his condition deteriorated, and he presented with an increased platelet count and further multiple complaints. Romiplostim was discontinued, and he was continued on prednisone 20 mg daily, after which he improved, and his platelet count reduced to 273,000 on 20 mg prednisone. This case calls attention to the need to review the role of combination therapy in treating refractory ITP and the prevention of complications of thrombocytosis secondary to advanced therapy. Treatment needs to be more streamlined, focused, and goal-directed. Escalation and de-escalation of treatment should be synchronized to prevent adverse complications from overtreating or undertreating.

## Introduction

Immune thrombocytopenia (ITP) is an isolated platelet count of <100,000/micro caused by autoimmune antibodies directed against platelet antigens. ITP can be due to primary autoimmune mechanisms or secondary to other conditions [1,2]. Refractory ITP is defined as ITP that does not respond to or relapse after splenectomy [2]. Clinical manifestations of ITP include fatigue, petechiae, purpuric lesions, epistaxis, gastrointestinal bleeding, and cerebrovascular hemorrhage. ITP is a diagnosis of exclusion [3]. Refractory ITP is becoming a subject of interest as there appears to be treatment failure and resistance to modern conventional treatment, necessitating a more universal and goal-directed approach to management. The effective roles of advanced therapies in the management of refractory ITP remain challenging.

## Case presentation

**Figure 1:**
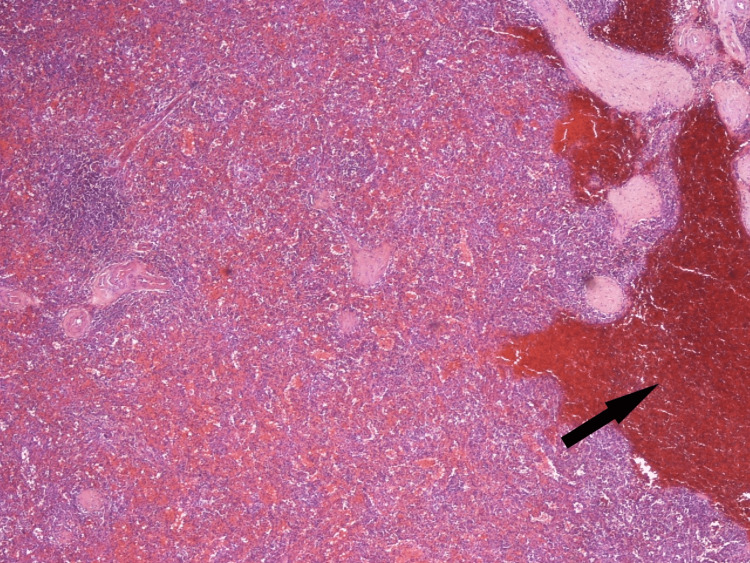
Benign spleen Arrow indicates the focal area of intraparenchymal hemorrhage and rupture (x40).

**Figure 2:**
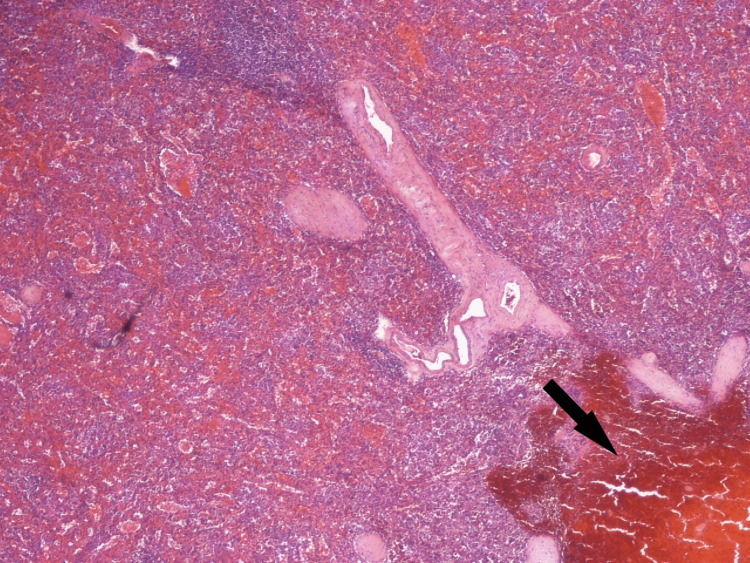
Benign spleen (225 grams) Arrow indicates the focal area of intraparenchymal hemorrhage and changes compatible with immune thrombocytopenia (x40).

**Figure 3:**
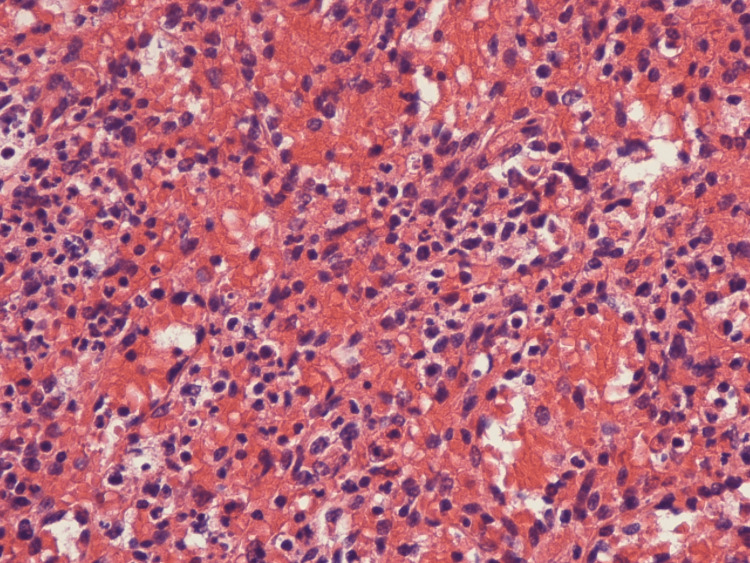
Unremarkable red pulp (red cells/area; x400)

**Figure 4:**
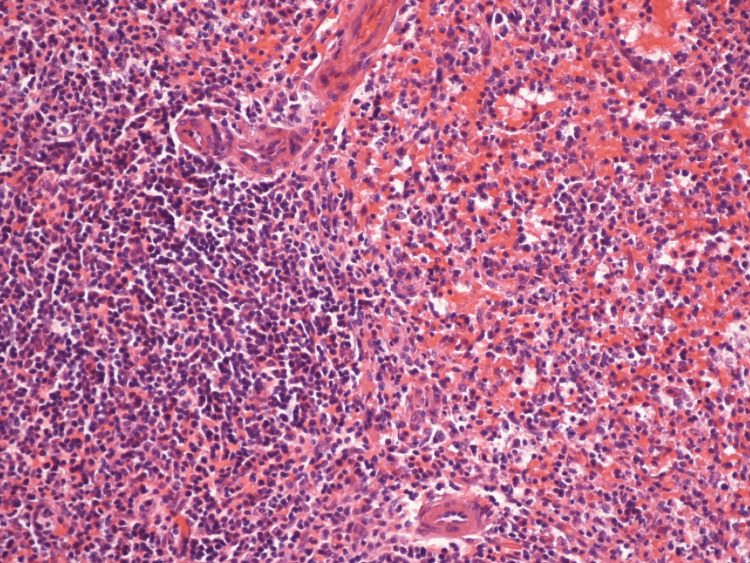
Hyperplasia of white pulp (blue cells; x200) Unremarkable white and red pulp excludes other causes of thrombocytopenia and confirms the diagnosis of immune thrombocytopenia.

Our patient is a 74-year-old male who presented to the emergency department (ED) with complaints of melena stools and severe fatigue lasting for two days. He was diagnosed with primary ITP six years before presentation, during a preoperative workup for his head and neck cancer, and has received multiple lines of treatment, including multiple courses of pulsed dexamethasone/prolonged courses of prednisone, numerous rounds of platelet transfusion, intravenous immunoglobulin, rituximab, and eltrombopag. About six weeks prior to index admission, he presented with fatigue and epistaxis. A peripheral blood smear revealed overt thrombocytopenia with normal platelet size and no platelet clumping. The treatment guideline was discussed with the patient and he opted for splenectomy. The spleen pathology showed a benign enlarged spleen (225 grams) with a focal area of intraparenchymal hemorrhage/rupture and changes compatible with ITP (Figures 1-4).

On index admission, his initial evaluation was significant for a petechial rash over extremities and bruises on upper extremities. Laboratory investigations showed decreased platelet count, decreased hemoglobin, and raised white blood cell count (Table 1).

**Table 1:** Laboratory investigations findings of the patient

Test	Finding	Reference range
White blood cell count (x1,000/UL)	21.0	5.0-10.0
Hemoglobin (mg/dl)	8.3	14.0-18.0
Platelet count (x1,000/UL)	8	150-400

Our patient expressed anger and frustration after realizing his ITP is refractory to splenectomy; he initially refused treatment; however, we provided him optimal care during hospitalization with effective communication. He received an intravenous bolus of 250 ml of normal saline, fresh frozen plasma, two units of irradiated platelet, and one unit of a packed red blood cell. He was started on Protonix infusion in the ED. Post-transfusion, his platelet count dropped to 4,000, and hemoglobin dropped to 6.4; he reported melena stool, hematochezia, an episode of hematemesis, and hemoptysis. Gastroenterology was consulted, and upper gastrointestinal endoscopy was deferred until thrombocytopenia was resolved. He was managed with multiple platelet transfusions, intravenous methyl prednisone succinate, rituximab, and romiplostim. His platelet count improved to 47,000, and he was discharged home on oral steroids with outpatient hematology follow-up. In a few weeks, his platelet count increased to 508,000, with associated headaches, blurry vision, and fatigue. Romiplostim was discontinued, and he was continued on prednisone 20 mg daily. The follow-up platelet counts improved to 273,000 on 20 mg prednisone.

## Discussion

Refractory ITP treatment aims to achieve a safe platelet count to prevent clinically significant complications, and not to normalize the platelet count [4]. Advanced therapy for refractory ITP includes splenectomy, rituximab, thrombopoietin receptor agonist (TPO-RA), and other immunosuppressive agents. All treatments effectively increase the platelet counts in most patients; however, each therapy varies in adverse effects [5,6]. Splenectomy is the most likely of all treatments to result in a cure or extended-lasting response [7]. The 2019 American Society of Hematology guideline makes a weak recommendation for TPO-RA over rituximab and for rituximab over splenectomy, but the guideline emphasizes that therapy should minimize toxicity and optimize the patient’s quality of life; as a result, the choice of management is highly dependent of patient values and preferences [6]. Our patient chose splenectomy; however, he developed severe thrombocytopenia with gastrointestinal tract bleeding two months postoperatively, necessitating treatment with combination therapy (intravenous steroid, rituximab, and romiplostim). The synergistic effects of combination therapy increase the risk for adverse outcomes compared to patients treated with a single treatment.

The use of corticosteroids as first-line therapy for adult ITP has been established over decades using oral prednisone at 1 mg/kg/d, slowly tapering to the lowest possible dose and titrating with the platelet count during weeks [8]. Other steroids such as dexamethasone, intravenous immunoglobulin, rituximab, eltrombopag, and surgical intervention of splenectomy subsequently find a place as therapy for adult ITP, as stated in the American Society of Hematology 2019 guidelines for ITP [6]. Although, consequently, the various agents used have their corresponding side effects. When combined, the synergistic effects of combination therapy increase the risk for adverse outcomes compared to patients treated with a single treatment.

The index patient received multiple lines of treatment as at diagnosis. He received multiple pulsed dexamethasone/prolonged courses of prednisone, numerous rounds of platelet transfusion, intravenous immunoglobulin, rituximab, and eltrombopag, and subsequently had a splenectomy. With a resolution of symptoms, he relapsed six years following first-line therapy. Several cases of relapse have been reported by different authors, with a patient having one to nine episodes of relapse [9-13]. Therefore, refractory ITP is not only associated with the burden of prolonged therapy's side effects but also a risk of hospital readmission, infection from immunosuppression, life-threatening complications such as upper or lower GI bleeding, stroke, epistasis, and psychological problems such as depression [14]. Our patient expressed anger and frustration toward the care team after realizing his ITP is refractory to splenectomy; he initially refused treatment; however, we could provide him optimal care during hospitalization with effective communication. A systematic review of splenectomy for ITP that included data for 47 case series (2623 adults) and over 50 years of observation, was published in 2004. It reported the only clinical parameter that predicts a favorable response to splenectomy is patient age. Younger patients have a higher response rate, although a specific age cut-off below which splenectomy was more effective could not be determined.

The index case is similar to a report by the American Society of Hematology [15]. Their patient was initially treated with high-dose steroids by the hematologist. With a short duration of response, she required multiple additional lines of therapy to maintain a platelet count above 30,000/µL, including splenectomy, rituximab, eltrombopag, and immunosuppression. However, she had a relapse three years after the initial treatment. In as much there is a significant improvement in treatment with steroids, splenectomy, rituximab, eltrombopag, and immunosuppression, there is still evidence of relapse after these therapies; therefore, there is a need to develop a regimen with long-standing remittance.

With the increasing rate of relapse and complications of steroid use, in the past few years, there has been a gradual shift from immune suppression for the treatment of ITP. With the advent of the COVID-19 pandemic, immune suppression became unrewarding as a treatment modality, and the focus has been on minimizing patient risk from COVID-19 [16]. The TPO-RAs are becoming more valuable as the first-line therapy to avoid immune suppression. Understanding the underlying pathophysiology of the disorder has helped develop several new targeted therapies, which include inhibitors of the neonatal Fc receptor inhibitors, Bruton tyrosine kinase, and complement pathway [17]. However, more studies and surveillance are needed to support the efficacy and longtime remission.

Regardless of therapy, all patients with ITP should be monitored regularly, and patients should be advised to consult their physician for any signs of bleeding, thrombosis, and infection. There is a limited established guideline in the management of refractory ITP after a failed initial treatment course with corticosteroids, intravenous immunoglobulin, eltrombopag, and splenectomy. This case calls attention to the need to review the role of combination therapy in treating refractory ITP and preventing complications. Several treatments exist for the management of ITP, and treatment algorithms must be centered on individualized care as patient characteristics and clinical response vary significantly. The need to monitor patients closely cannot be overemphasized, as some patients can develop bleeding, stroke, and thrombocytosis following treatment with advanced therapies. Our patient developed symptomatic thrombocytosis leading to treatment discontinuation to restore normal platelet counts.

## Conclusions

Treatment needs to be more streamlined, focused, and goal-directed. In addition, escalation and de-escalation of treatment should be synchronized to prevent adverse complications from overtreating or undertreating. Combination therapies still remain the cornerstone of managing refractory ITP and it is highly recommended that physicians identify patients who failed multiple treatments for ITP early before irreversible complications become established.
